# Chlorine disinfection facilitates natural transformation through ROS-mediated oxidative stress

**DOI:** 10.1038/s41396-021-00980-4

**Published:** 2021-05-03

**Authors:** Shuai Zhang, Yue Wang, Ji Lu, Zhigang Yu, Hailiang Song, Philip L. Bond, Jianhua Guo

**Affiliations:** 1 Advanced Water Management Centre (AWMC), The University of Queensland, St Lucia, Brisbane, QLD Australia; 2 Jiangsu Key Laboratory of Atmospheric Environment Monitoring and Pollution Control, Collaborative Innovation Center of Atmospheric Environment and Equipment Technology, Nanjing University of Information Science & Technology, Nanjing, China; 3 School of Environment, Nanjing Normal University, Jiangsu Engineering Lab of Water and Soil Eco-remediation, Nanjing, China; 4 Center for Microbiome Research, School of Biomedical Sciences, Queensland University of Technology, Brisbane, QLD Australia

**Keywords:** Antibiotics, Public health

## Abstract

The bacterial infection that involves antimicrobial resistance is a rising global threat to public health. Chlorine-based water disinfection processes can inactivate antibiotic resistant bacteria. However, at the same time, these processes may cause the release of antibiotic resistance genes into the water as free DNA, and consequently increase the risk to disseminate antibiotic resistance via natural transformation. Presently, little is known about the contribution of residual chlorine affecting the transformation of extracellular antibiotic resistance genes (ARGs). This study investigates whether chloramine and free chlorine promote the transformation of ARGs and how this may occur. We reveal that both chloramine and free chlorine, at practically relevant concentrations, significantly stimulated the transformation of plasmid-encoded ARGs by the recipient *Acinetobacter baylyi* ADP1, by up to a 10-fold increase. The underlying mechanisms underpinning the increased transformations were revealed. Disinfectant exposure induced a series of cell responses, including increased levels of reactive oxygen species (ROS), bacterial membrane damage, ROS-mediated DNA damage, and increased stress response. These effects thus culminated in the enhanced transformation of ARGs. This promoted transformation was observed when exposing disinfectant-pretreated *A. baylyi* to free plasmid. In contrast, after pretreating free plasmid with disinfectants, the transformation of ARGs decreased due to the damage of plasmid integrity. These findings provide important insight on the roles of disinfectants affecting the horizontal transfer of ARGs, which could be crucial in the management of antibiotic resistance in our water systems.

## Introduction

The dissemination of antimicrobial resistance poses a serious worldwide threat to public health [1]. Horizontal gene transfer (HGT) is recognized as one of the major drivers for disseminating antibiotic resistance genes (ARGs) [2]. HGT will occur by conjugation, transduction, and transformation of DNA [3]. Conjugation is the transfer of DNA between a donor and a recipient, which is usually mediated via mobile genetic elements in bacteria, such as plasmids, transposons, and integrons [3]. Transduction occurs when DNA is transferred to other bacteria through bacteriophage infection [2]. Transformation results from the uptake and incorporation of extracellular DNA directly through the bacterial cell membranes [4]. harboring ARGs from the surroundings through bacterial cell membranes [4]. Either of these HGT processes can result in an enhanced spread of ARGs. For natural transformation to occur, bacterial cells must become competent for DNA uptake [5]. Unlike conjugation or transduction, viable donor bacteria are not needed for transformation [6].

Although antibiotic resistance is of major concern worldwide, the spread of antibiotic resistance in water systems has been overlooked to date [7–9]. In particular for role of how water disinfection may influence the HGT of ARGs is unknown. Water disinfection is of paramount importance to the quality of water supply and to human health. Chlorination is a widely used disinfection treatment of water or wastewater for the removal of pathogens and potentially antibiotic resistant bacteria (ARB) and ARGs [10–13]. However, commonly used chlorine disinfectants are less effective on highly chlorine resistant waterborne bacteria, such as pathogenic *Nontuberculous mycobacteria* and *Pseudomonas aeruginosa*, can exist in drinking tap water and thwart the disinfection process [14, 15]. Moreover, the disinfection processes may inactivate or damage ARB, and result in the release of ARGs into the water. This would generate increased extracellular ARGs (eARGs) in the environment that would be available for spread by transformation [16, 17]. Recently it was observed that the abundances of both intracellular ARGs (iARGs) and eARGs were enhanced after chlorine disinfection in a full-scale wastewater treatment plant (WWTP) [18]. The occurrence of iARGs may promote ARB dissemination via conjugation and transduction. In contrast, eARGs persisting in the aquatic environment can be taken up by competent bacteria and cause the dissemination of antibiotic resistance through transformation [6, 19]. Such unexpected observations [18] inspired the hypothesis that disinfectant agents could play a key role in the dissemination of eARGs via transformation in water treatment systems.

This study aims to reveal whether and how residual disinfectants (both chloramine and free chlorine) facilitate the spread of eARGs through transformation. We developed three transformation systems to investigate whether these disinfectants promoted the transformation efficiency of eARGs in a drinking water distribution system and in the effluent of a WWTP. The bacterial transformation system comprised of the plasmid, pWH1266, carrying *bla*_TEM-1_ and *tetA* as the eARGs, and the recipient bacteria *Acinetobacter baylyi* ADP1. The mechanisms underpinning the increased transformations were revealed by assessing oxidative stress and cell membrane permeability by flow cytometry, by observing plasmid damage detected with atomic force microscopy (AFM), as well as by evaluating molecular responses detected through qPCR, genome-wide RNA sequencing, and proteomic sequencing. These findings advance our understanding of the role of the disinfection process on the dissemination of antibiotic resistance and suggest that disinfection may need to be carefully optimized, ensuring it does not contribute to the spread of antibiotic resistance.

## Materials and methods

### Bacterial strains and culture media

The plasmid pWH1266 (Fig. S1, 8.89 kb, ATCC 77092) was selected as the free plasmid DNA. The plasmid pWH1266 carries two ARGs (*tetA* and *bla*_TEM-1_) against tetracycline (Tet) and ampicillin (Amp), respectively. The host *Escherichia coli* cells harboring the plasmid pWH1266 were grown overnight in 20 mL Lysogeny broth (LB, Sigma-Aldrich, pH of 7.4) containing 50 mg/L Amp at 37 °C shaken at 150 rpm. The plasmid within the host *E. coli* cells was extracted using the Invitrogen PureLink Quick Plasmid Miniprep Kit (Life Technologies, USA). The presence of the plasmid was detected by 1% agarose gel electrophoresis and its concentration was determined on a NanoDrop (Thermo Scientific, Waltham, MA).

*A. baylyi* ADP1, a naturally transformable Gram-negative bacterium [20], was chosen as the recipient. ADP1 was grown in LB broth, shaken at 30 °C overnight (140 rpm). Subsequently, the cell suspension was diluted 100 times in LB broth and incubated for 6 h, shaken at 140 rpm at 30 °C, to reach an OD600 of 1.1. The recipient cells were pelleted by centrifugation at 6000 × *g* for 5 min. The supernatants were removed and the pellets were washed twice in phosphate-buffered saline (1 × PBS, pH = 7.2), and resuspended in PBS. Tet and Amp were purchased from Sigma-Aldrich (USA) and Amp Gold Biotechnology (USA) respectively.

### Transformation model systems and transformation assays

To evaluate the effects of chlorine-based disinfectants on transformation frequency, we established three transformation model systems. System 1 was established to evaluate whether the residual disinfectants could facilitate the spread of eARGs through transformation. Specifically, 500 μL transformation systems comprising of the recipient bacteria at 10^8^ cfu/mL and the free plasmid at 0.8 ng/μL were established. The systems were exposed to chloramine or free chlorine at concentrations of 0, 0.5, 2, 4, 10, 20, and 30 mg/L. Free chlorine was prepared by diluting sodium hypochlorite (5%, Sigma, USA) in PBS solution. Chloramine was prepared through the addition of sodium hypochlorite and ammonium chloride as previously described [19, 21]. In this method, a mass ratio of 4:1 of chlorine to nitrogen was used to obtain the chloramine. The transformation assays were conducted under conditions mimicking drinking water which were performed in a PBS solution without shaking, and no chemical oxygen demand (COD) was added. As well, transformation assays were conducted under conditions mimicking a WWTP effluent containing 50 mg/L of COD by the addition of sodium acetate, and without shaking. This COD concentration is typical of that detected in WWTP effluents [22].

System 2 was set up to investigate whether the transformation of eARGs increased when only *A. baylyi* stains were treated by disinfectants first and intact plasmids were added later for transformation. Specifically, 500 μL of the wild-type *A. baylyi* (10^8^ cfu/mL) were exposed to chloramine or free chlorine at concentrations of 0, 0.5, 2, 4, and 10 mg/L. After 5 or 15 min pretreatment by the residual disinfectants, the samples were centrifuged immediately (12,000 × *g*, 3 min) to pellet the cells. After discarding the supernatant, the cell pellets were washed twice and resuspended in 500 μL PBS. These disinfectant-treated *A. baylyi* were then mixed with free pWH1266 plasmid at a final concentration of 0.8 ng/μL.

Another System 3 was set up to test whether the transformation of damaged eARGs would be decreased if the naked plasmid DNA was pretreated by residual disinfectants. Specifically, 500 μL solutions of purified plasmids (0.8 ng/μL) were exposed to chloramine or free chlorine at the concentrations of 0, 0.5, 2, 4, and 10 mg/L for either 5 or 15 min. Then, 500 μL of ethyl alcohol (Sigma) was added into the treated plasmid solution. The treated plasmids were then applied in transformation assays to measure the ARGs transformation efficiency in conditions mimicking WWTP effluent. In brief, 475 μL of the *A. baylyi* strains were combined with 25 μL of treated or untreated plasmid at 0.8 ng/μL. The same samples were assayed by qPCR to quantify the loss of ARGs as described below.

For these three systems, the mixture of the plasmid and the recipient was incubated at 25 °C for 6 h. Serial dilutions of the suspensions were taken and spread onto selective LB media plates containing 100 μg/mL of Amp and 5 μg/mL of Tet. The plates were then incubated at 30 °C for 48 h and colonies of transformants were counted. In addition, the total recipient counts were determined by spreading the mixture suspensions onto LB plates that did not contain antibiotics. The transformation frequency was calculated by normalizing the total number of transformants to the total number of recipients.

### Transformation assays under anaerobic conditions

Experiments were conducted using the transformation model System 1 in an anaerobic chamber under anaerobic conditions (details in Supporting Information Text S1). Briefly, this was to verify whether ROS were implicated in the transformation of eARGs. The anaerobic transformation assays were performed in the same manner as described above for aerobic conditions, except that oxygen in LB or PBS solutions was depleted and the experiments were conducted in an anaerobic chamber (Coy Laboratory Products Inc., USA).

### Measurements of minimum inhibitory concentrations (MICs) and residual chlorine

The MICs of chloramine, free chlorine, Amp, and Tet, against the recipient *A. baylyi* and transformants were determined as previously described [23]. In detail, the bacteria were grown and diluted to ~10^5^ cfu/mL. To each well within the 96-well plates, 5 μL of the bacterial culture, 15 μL of disinfectants or antibiotics (with different concentrations), and 130 μL of fresh LB media were added. The plates were incubated at 30 °C for 20 h before the OD_600_ was measured on the plate reader (Tecan Infinite M200, Switzerland). MICs of the bacterial strains were determined as the concentration of disinfectants or antibiotics which inhibited 90% of the growth. Each bacterial strain under the inhibition of the disinfectants or antibiotics was tested at least in triplicate.

The concentrations of residual chlorine were obtained using the Pocket Colorimeter II analysis system (Hach company, Beijing, China).

### Detection of reactive oxygen species (ROS) and cell membrane permeability

After exposure to chloramine or free chlorine, both intracellular ROS production [24] and cell membrane permeability of the recipient *A. baylyi* were measured using a CytoFLEX flow cytometer (Beckman Coulter, USA) following the previously described procedure [25]. For System 2, the intracellular ROS generation was also measured for the disinfectant-treated *A. baylyi* (details are shown in Supporting Information Text S2). In addition, ROS production and cell membrane permeability were detected when exposing recipient *A. baylyi* to disinfectants under anaerobic conditions (details are shown in Supporting Information Text S3).

### Electron paramagnetic resonance (EPR) spectroscopy characterization

EPR spectroscopy was employed to detect radicals generated during the disinfectant treatment. This was performed on a Bruker ElexsysE500 (Billerica MA) at room temperature. A spin-trapping agent, 5,5-dimethyl-1-pyrroline-N-oxide (DMPO), at a concentration of 50 mM was mixed with *A. baylyi* in either aerobic or anaerobic conditions. Then, chloramine or free chlorine, at final concentrations of 30 mg/L, were added to the mixture. After 5 min, samples of the mixture were transferred into a capillary tube, which was then fixed into the cavity resonator and the EPR spectra was recorded. Mixtures in the absence of disinfectants were analyzed as controls. For the EPR analysis the modulation frequency of 100 kHz, the band microwave of 9.43 GHz, at 1.5 mW, and a sweeping magnetic field of 110 K were used [26, 27].

### Quantification of live and dead cells

The inhibitory effects of disinfectants on recipient bacteria *A. baylyi* in aerobic and anaerobic conditions were determined by evaluating the live to dead cell proportions. This was conducted using the BacLight Bacterial Viability Kit (Invitrogen, USA). In brief, overnight grown cells were exposed to the disinfectants as described for the different treatment Systems and samples were taken and dual stained with propidium iodide (PI, final concentration: 30 μM) and SYTO 9 (final concentration: 5 μM). A CytoFLEX flow cytometer (Beckman Coulter, USA) was used to detect the fluorescence. The PI-positive cells were recorded as dead, while the SYTO 9-positive cells were live.

### Plasmid extraction, PCR, and gel electrophoresis

In order to verify that the colonies on the antibiotic plates carried the pWH1266 plasmid, transformants (at least six colonies from each treatment condition) were randomly selected and cultured in LB broth overnight. Plasmids of the transformants were extracted using the InvitrogenTM PureLink Quick Plasmid Miniprep Kit. The *tetA* and *bla*_TEM-1_ genes were detected with both short and long amplicon PCR (Bio-Rad C1000 Touch, USA) (the primers are listed in Supporting Information Table S1). Each 25 µL PCR reaction contained 12 µL of Master Mix (Thermo Fisher Scientific, USA), 0.8 µL of forward and reverse primers (0.4 μL of 100 μM each), 0.4 µL of DNA templates, and 11.8 µL of sterile ddH_2_O. The presence of plasmids and ARGs were detected using 1% agarose gel electrophoresis.

### Quantification of ARG by real-time qPCR

The *tetA* and *bla*_TEM-1_ genes carried by the pWH1266 plasmid were quantified by detection of short and long amplicons with a Real-Time PCR System. The primer sequences and qPCR protocol conditions were performed as previously described [6]. Briefly, each 10 µL qPCR reaction contained 5 µL of SYBR Green Master Mix, 0.6 µL of forward and reverse primers (0.3 μL of each at 100 μM), 0.2 µL of DNA template, and 4.2 µL of sterile ddH_2_O. Standard curves were conducted in triplicate (Regression coefficients, *R*^2^ > 0.990). The temperature profile included one cycle at 95 °C for 2 min, then 40 cycles of the steps that included 95 °C for 10 s, an annealing temperature (Table S1) for 20 s, and finally 72 °C for either 60 s (long amplicons) or 20 s (short amplicons). The amplification efficiencies for *tet*A and *bla*_TEM-1_ genes were greater than 0.70 ± 0.06.

### Transmission electron microscopy (TEM) and AFM

The cell morphology of *A. baylyi* was determined by TEM to visualize whether the cell membranes were affected by exposure to chloramine or free chlorine under conditions mimicking drinking water in System 1. This was performed as described previously [28] where *A. baylyi* was exposed to 10 mg/L of chloramine or free chlorine for 6 h. Samples of the cell mixture were taken and prepared for visualization.

The surface topography of dissociative plasmid pWH1266 DNA in System 3 was characterized using a Cypher AFM (Asylum Research, Oxford Instruments, CA). The experiments were conducted by exposing 500 μL solutions of purified plasmids (0.8 ng/μL) to chloramine or free chlorine at the concentrations of 0, 4, and 10 mg/L for 15 min. Samples were then taken for the AFM characterization where the DNA deposition was conducted using the spinning method [29].

### RNA extraction, genome-wide RNA sequencing, and bioinformatics

Samples were taken from the transformation System 1, mimicking WWTP effluent (PBS with 50 mg/L COD), after 2 h exposure to chloramine or free chlorine at 0 mg/L (control) or at 10 mg/L. The total RNA was extracted using the RNeasy Mini Kit (QIAGEN, Germany) from three samples of the control experiment, three samples from the chloramine-treated group and three samples of the chlorine-treated group. The extracted RNA was used for strand-specific cDNA library construction and Illumina paired-end sequencing (HiSeq 2500, Illumina Inc., San Diego, CA) in Macrogen Co. (Seoul, Korea). The mRNA expression levels were normalized as fragments per kilobase of exon model per million reads mapped (FPKM). A bioinformatic pipeline was applied to evaluate the mRNA expressions as described in our previous study [30].

### Protein extraction and proteomic analysis

Protein extractions were performed on samples taken from another transformation System 1 experiments using LB broth as the medium after 6 h exposure to chloramine or free chlorine at 10 mg/L. Cells were harvested by centrifugation (12,000 × *g* centrifugation for 10 min) from three samples of the control experiment (no disinfectant exposure), from three samples of the chlorine treatment at 10 mg/L, and three samples of the free chlorine treatment at 10 mg/L. These pelleted cells were then subjected to protein extraction and proteomic bioinformatics analyses as described previously [23, 31].

### Statistical analysis

Significant differences were performed by one-way ANOVA test, and *p* values were corrected using the Benjamini–Hochberg method and presented as *p*_*adj*_ [32]. A value of *p*_*adj*_ < 0.05 was considered statistically significant. Data were expressed as the mean ± standard deviation.

## Results

### Disinfectants promote transformation frequency

**Fig. 1:**
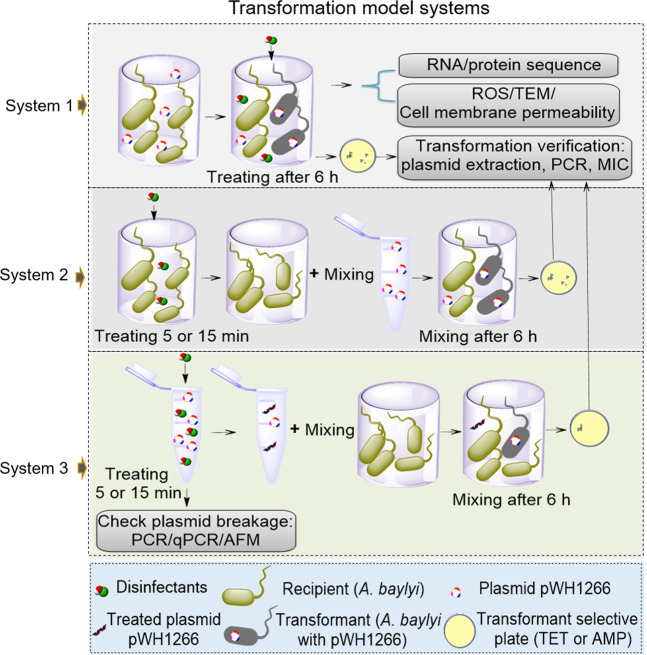
Schematic depicting experimental design and methodologies. Three transformation model systems were established. Oxidative stress and cell membrane permeability, RNA sequencing and proteomic sequencing were assessed to reveal underlying mechanisms in System 1.

To examine the effects of chlorine-based disinfectants on the spread of eARGs, we investigated the transformation frequency of the pWH1266 plasmid under the exposure of chloramine or free chlorine by establishing three systems (Fig. 1). The exposure concentrations of chloramine and free chlorine we used are relevant to disinfectant dosages in drinking water (0–4 mg/L) and secondary effluent from WWTP (1–20 mg/L) [19, 33]. In addition, an extremely high concentration (30 mg/L) was also applied in this study to test the high chlorine resistance of *A. baylyi* in terms of MIC analyses (Table S2). The transformation efficiency of eARGs was evaluated based on the experimental design and methodologies as illustrated in Fig. 1. First, the transformation system conditions were optimized for the initial plasmid concentration and for the transformation time. The frequency of eARGs transformation was ~1.5 × 10^−7^ after 6 h (Fig. S2), and was maximized when the plasmid concentration was greater than 0.8 ng/μL (Fig. S3). Therefore, we employed 0.8 ng/μL as the initial plasmid concentration and 6 h as the transformation time for all the transformation assays in this study.

**Fig. 2:**
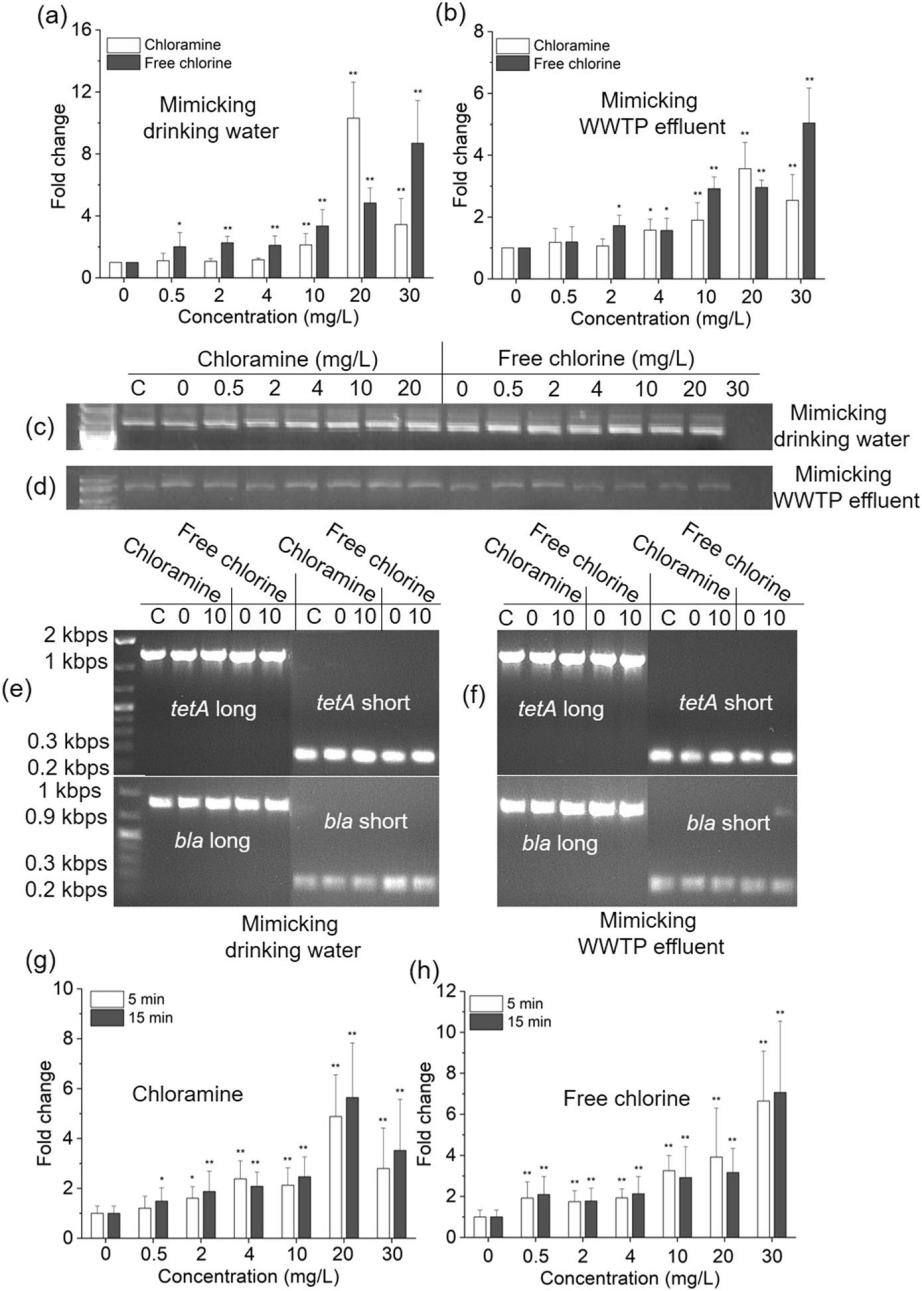
Phenotypic results of the transformation of ARGs induced by different concentrations of disinfectants. **a** Effects of disinfectants on the transformation frequency of ARGs in mimicking drinking water. **b** Effects of disinfectants on the transformation frequency of ARGs in mimicking downstream of WWTP effluent. Electrophoresis gel of plasmid pHW1266 extracted from transformants (Transformation system treated with 0 (control), 0.5, 2, 4, 10, 20, 30 mg/L chloramine or free chlorine, respectively) in mimicking drinking water (**c**) and in mimicking downstream of WWTP effluent (**d**). Electrophoresis gel of *tetA* and *bla*_TEM-1_ measured with PCR short amplicon (SA) and long amplicon (LA) extracted from transformants (Transformation system treated with 0 (control) and 10 mg/L chloramine or free chlorine, respectively) in mimicking drinking water (**e**) and in mimicking downstream of WWTP effluent (**f**). *A. baylyi* strains were pretreated by disinfectants for 5 min (**g**) and 15 min (**h**) caused increases in transformation frequency, respectively. Significant differences between individual chloramine or free chlorine and the control were shown with *(*p*_*adj*_ < 0.05) and **(*p*_*adj*_ < 0.01).

Both chloramine and free chlorine could significantly increase the number of transformants and the transformation frequency of plasmid-encoded eARGs in System 1 (Fig. 2a, b, and Fig. S4). Specifically, the transformation frequency of eARGs under conditions mimicking drinking water was significantly increased when dosages of chloramine or free chlorine were greater than 10 and 0.5 mg/L respectively (Fig. 2a, *p*_*adj*_ = 8.8 × 10^−4^–4.6 × 10^−2^). On the other hand, under conditions mimicking WWTP effluent, chloramine or free chlorine concentration had to be greater than 4 and 2 mg/L respectively, for the transformation frequency of eARGs to significantly increase (Fig. 2b, *p*_*adj*_ = 2.6 × 10^−6^–4.6 × 10^−2^). Moreover, the transformation frequency of eARGs exhibited a dosage-dependent trend with increased exposure to free chlorine. The maximum transformation frequency (2.6 × 10^−6^ per recipient cell) after adding 20 mg/L chloramine was more than 10 times greater than that of the control under conditions mimicking drinking water (Fig. 2a, *p*_*adj*_ = 1.4 × 10^−3^). However, the transformation frequency decreased when further increasing the chloramine concentration from 20 to 30 mg/L (Fig. 2a, b). In addition, concentrations of chlorine residuals were measured during the transformation experiment, indicating both chloramine and free chlorine dissipated with time due to disinfection reactions. For instance, concentrations of chlorine residuals decreased to 2.7 or 1.4 mg/L after 6 h of treatment when initial levels of 30 mg/L of chloramine or free chlorine were applied respectively (Fig. S5).

MIC testing, plasmid extraction, PCR and gel electrophoresis all confirmed that the pWH1266 plasmid had been uptaken by *A. baylyi* in System 1. The presence of the pWH1266 plasmid in transformants was confirmed by gel electrophoresis as clear bands were observed having the size approximating that of the plasmid from the donor (Fig. 2c, d). Secondly, the presence of *tetA* genes and *bla*_TEM-1_ genes in the randomly picked transformants tested positive with both the short and long amplicon PCR. All the *tetA* genes and *bla*_TEM-1_ genes exhibited clear bands with an approximate size of those present in the pWH1266 plasmid (Fig. 2e, f). This also supports that the transformants harbored the eARGs. Additionally, the MICs of transformants also supported that they had acquired eARGs against the two antibiotics (Amp and Tet). All the transformants exhibited increased resistance against the two antibiotics (Table S2), indicating the expression of the Amp and Tet resistance genes after uptake of the pWH1266 plasmid. In contrast, the wild-type *A. baylyi* was susceptible to antibiotics Amp and Tet (Table S2).

We further investigated whether the transformation of eARGs would be increased if the wild-type *A. baylyi* strains were first pretreated with residual disinfectants by employing System 2 experiments. Similarly, the transformation frequency of eARGs increased with increasing disinfectant doses (Fig. 2g, h). For instance, after treating *A. baylyi* strains with 20 mg/L chloramine for 5 min, the transformation frequency was ~4.9 times greater than that of the control group (Fig. 2g, *p*_*adj*_ = 1.2 × 10^−4^–2.4 × 10^−2^). When the pretreatment time was increased to 15 min, the transformation frequency was significantly increased by 5.6 times (Fig. 2g, *p*_*adj*_ = 7.6 × 10^−5^–1.0 × 10^−2^). After treating *A. baylyi* with free chlorine, the transformation frequency of eARGs also significantly increased when its concentrations were greater than 0.5 mg/L (Fig. 2h, *p*_*adj*_ = 4.1 × 10^−3^–5.5 × 10^−2^).

Collectively, these results demonstrated that disinfectants could significantly increase the transformant number and transformation frequency of eARGs in both Systems 1 and 2. The generated transformants indeed acquired resistance against Amp and Tet by uptaking pWH1266 plasmid. It should be noted that the transformation frequency might be overestimated if vertical gene transfer (VGT) occurred during the transformation period. However, this overestimation can be ruled out, since the total number of recipients did not increase during the 6-h period (Fig. S4 and Table S3), this indicating that the increased frequency was facilitated by transformation, rather than VGT.

### Disinfectants induce the increased generation of ROS and stimulate the stress response

**Fig. 3:**
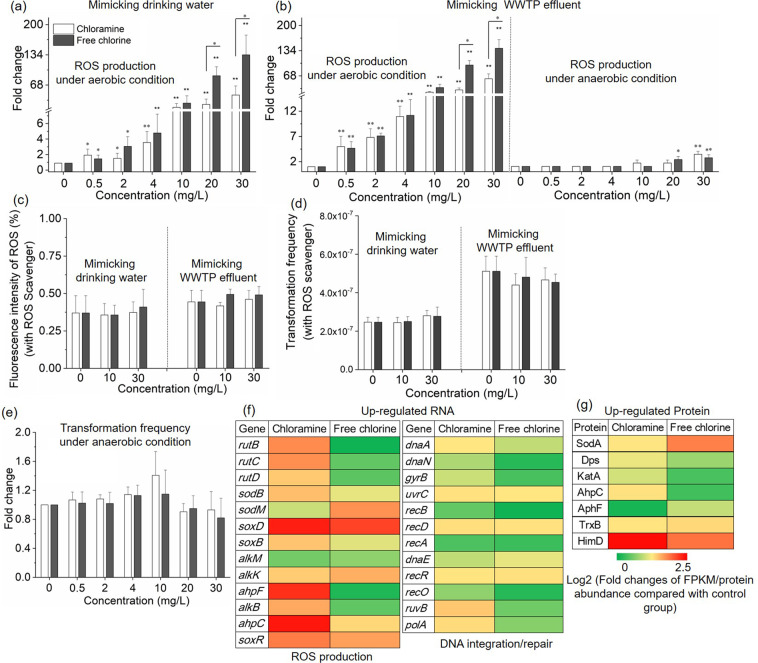
Effects of disinfectants on ROS generation and DNA integration/repair in the recipient bacterial strains. **a** Effect of disinfectants on the ROS generation in mimicking drinking water. **b** Effect of disinfectants on the ROS generation in mimicking downstream of WWTP effluent. **c** Effect of ROS scavenger on disinfectants-induced ROS generation. **d** Transformation frequency with the addition of ROS scavenger thiourea. **e** Fold change of transformation frequency under anaerobic condition. **f** Fold changes of expression of core genes related to ROS production and DNA integration/repair. **g** Fold changes of the abundance of core proteins related to ROS generation. For **a**–**e**, significant differences between individual chloramine or free chlorine and control samples were shown with *(*p*_*adj*_ < 0.05) and **(*p*_*adj*_ < 0.01).

It is seen that subinhibitory concentrations of antibiotics can increase the transformation of ARGs due to oxidative stress [34]. Here, we hypothesize that exposure to the two chlorine-based disinfectants caused increased production of ROS, and this promoted the transformation frequency of eARGs. Indeed, the recipient cells showed a significant increase of intracellular ROS generation under chloramine or free chlorine exposure from 0.5 to 30 mg/L under conditions mimicking both drinking water (Fig. 3a, *p*_*adj*_ = 7.7 × 10^−6^–4.3 × 10^−2^) and WWTP effluent (Fig. 3b, *p*_*adj*_ = 4.0 × 10^−6^–9.8 × 10^−3^). The maximum ROS production was detected during exposure to free chlorine at 30 mg/L. This was more than a 130-fold increase of ROS levels in comparison to the no-exposure control group. In addition, during exposure to the disinfectants, ROS production was greater in the conditions mimicking the WWTP effluent in comparison to the experiments mimicking drinking water. For example, the ROS levels under exposure to 4 mg/L chloramine in the WWTP effluent experiment were increased by 4.7-fold in comparison with levels detected in the simulated drinking water (*p*_*adj*_ = 1.2 × 10^−3^). This may be resulted from the addition of organic matter (50 mg COD/L) causing increased bacterial metabolic activity [35], and resulting in higher ROS production.

In order to further verify the role of increased ROS levels for enhancing transformation frequency, we conducted ROS scavenging tests. Thiourea, which is a typical ROS scavenger [36], was added to the transformation system. After the addition of 400 μM thiourea, the ROS production levels during disinfectant exposure were significantly decreased to the ROS levels of the no-disinfectant control groups (Fig. 3c, *p*_*adj*_ = 7.7 × 10^−6^–7.9 × 10^−4^). Correspondingly, the transformation frequencies were also significantly (*p*_*adj*_ = 6.1 × 10^−6^–7.3 × 10^−3^) reduced to the level of the control groups (Fig. 3d).

To further clarify the role of ROS for contributing to the enhanced transformation, experiments were conducted under strict anaerobic conditions, where ROS would not be generated [37]. As expected, after exposure to chloramine or free chlorine the bacterial ROS levels decreased significantly in comparison with those detected under aerobic conditions (Fig. 3b, Fig. S6, *p*_*adj*_ = 4.1 × 10^−6^–1.5 × 10^−4^) to the levels of the non-disinfectant controls. Moreover, when running the transformation experiment anaerobically, no significant enhancement of transformation frequency was detected during exposure to chloramine or free chlorine (Fig. 3e, *p*_*adj*_ = 0.0–0.33). In addition, significant declines were observed when comparing the transformation frequencies in anaerobic conditions with those measured under aerobic conditions (*p*_*adj*_ = 2.3 × 10^−4^–0.02). Thus, the anaerobic experiments support the hypothesis that the enhanced transformation of eARGs is due to the increased bacterial ROS generation during exposure to the chloramine or free chlorine disinfectants.

In relation to oxidative conditions, generated radicals were characterized by EPR spin trapping with DMPO. Under aerobic conditions, both chloramine and free chlorine (30 mg/L) could generate the ClO• radical with strong intensity (Fig. S7). Anaerobically, the intensity of the DMPO-trapped radicals decreased [26]. Also, the radicals generated were different when exposing *A. baylyi* to these disinfectants anaerobically. For free chlorine, the major radical was still ClO•, while it turned to be a HO• radical under the exposure of chloramine. This may attribute to different modes of action of these two chlorine-based disinfectants against *A. baylyi*.

We further employed transcriptional analysis to provide molecular evidence that the exposure to the chlorine-based disinfectants at 10 mg/L triggered oxidative stress in the recipient strains (Fig. 3f, Table S4). Differentially transcribed oxidative stress genes between the chloramine or free chlorine-treated groups and the control were compared based on genome-wide RNA sequencing. Increased expression of oxidative stress-related genes indicated the recipient bacteria responded quickly to the disinfectant exposure. Indeed, after 2 h exposure to disinfectants the transcriptional levels of superoxide dismutase (*sodB* and *sodM*) were increased. For example, 1.3- and 2.4-fold (*p* < 0.05) increases of *sodM* genes were observed in the recipient bacteria in response to chloramine or free chlorine exposure respectively. The expression levels of *ahpC* (more than 4.9-fold under chloramine, *p* < 0.05) and *ahpF* (more than 4.3-fold under chloramine, *p* < 0.05), which code for alkyl hydroperoxide reductase [38], were upregulated under exposure to the disinfectants. In addition, *soxD*, coding for sarcosine oxidase was upregulated more than 4.7-fold and 3.8-fold in the pressure of chloramine or free chlorine respectively (*p* < 0.05). These antioxidant enzymes were likely overexpressed to protect the recipient from the damage caused by increased ROS levels [39]. In particular, the *soxR* gene in *A. baylyi*, which plays a key role in the ROS response, was upregulated more than 2.8-fold in the pressure of chloramine [40]. Furthermore, the expression of core genes related to DNA integration and repair showed increased transcript levels under the exposure of chloramine or free chlorine (Fig. 3f, Table S5). For example, after exposure of *A. baylyi* to chloramine or free chlorine, *gyrB*, the *rec* gene family (*recA*, *recB*, *recD*, *recO*, and *recR*) and *ruvB*, which are genes relevant to DNA integration and repair [16], showed increased expression levels.

Protein sequencing was further employed to examine the bacterial response to the oxidative stress associated with disinfectant exposure. The proteins such as SodA, KatA, Ahp, and HimD, which are relevant to increased ROS levels, were increased during the exposure to chlorine-based disinfectants (Fig. 3g). For example, 5.3- and 3.0-fold (*q* < 0.01) increases of HimD protein were observed in the recipient bacteria in response to chloramine or free chlorine exposure, respectively. In particular, SodA and Ahp family proteins (AhpC and AphF) are antioxidant enzymes that play a role in the protection from ROS attack [39], were upregulated (Table S6).

### Disinfectant exposure increased the cell membrane permeability

**Fig. 4:**
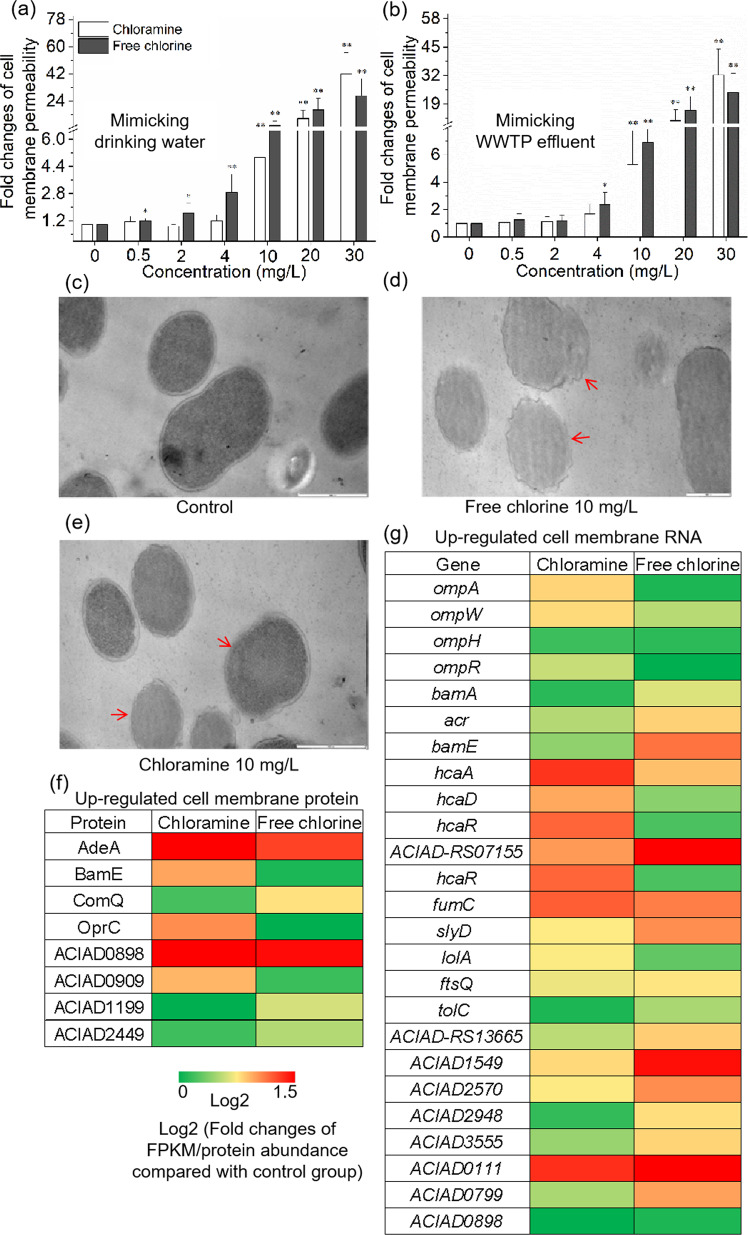
Effects of disinfectants on cell membrane in recipient *A. baylyi*. **a** Effects of disinfectants on the cell membrane permeability in mimicking drinking water. **b** Effects of disinfectants on the cell membrane permeability in mimicking downstream of WWTP effluent. TEM images of *A. baylyi* in ultrafine slices are shown for the untreated control group (**c**) and the cells treated with 10 mg/L of free chlorine (**d**) or chloramine (**e**) (scale bars are 1 μm). **f** Heat map showing the enhanced abundance of proteins related to cell membrane in the *A. baylyi*. **g** Heat map showing the upregulated genes related to cell membrane in the *A. baylyi*. For **a**, **b**, significant differences between individual chloramine or free chlorine and the control were shown with *(*p*_*adj*_ < 0.05) and **(*p*_*adj*_ < 0.01).

Membrane permeability is one of the barriers to the uptake of foreign or extracellular DNA. It is thought that an increased membrane permeability caused by pore formations on the cell surface can enhance transformation frequency [5, 41]. Thus, the cell membrane permeability of recipient bacteria was evaluated to verify whether the enhanced transformation was associated with the increased membrane permeability under the exposure to the disinfectants. Indeed, the recipient strain showed a significant increase of cell membrane permeability under chloramine or free chlorine (Fig. 4a, b, *p*_*adj*_ = 7.2 × 10^−6^–4.9 × 10^−2^). For example, cell membrane permeability during exposure to 30 mg/L of chloramine showed a 41.8-fold increase in comparison with the control group in the experiments mimicking drinking water (Fig. 4a, *p*_*adj*_ = 1.1 × 10^−5^). In the WWTP effluent experiments, significantly increased membrane permeability was also observed (Fig. 4b, *p*_*adj*_ = 4.3 × 10^−5^–4.9 × 10^−2^). However, the membrane permeability changes were slightly different between the drinking water and WWTP effluent experiments. In particular, the membrane permeability of the recipient in the WWTP simulated effluent began to enhance significantly (Fig. 4b, *p*_*adj*_ = 2.5 × 10^−2^) when the free chlorine concentration was equal to or greater than 4 mg/L. In contrast, significant permeability changes were detected in the simulated drinking water experiments at the lower level of 0.5 mg/L of chlorine. This phenomenon may be a result of the chlorine being consumed to oxidize organic matter (50 mg COD/L) added in WWTP effluent studies. In addition, it can be found that the threshold value of chlorine (above 0.5 mg/L for drinking water while above 4 mg/L for WWTP effluent) to cause the enhanced cell membrane permeability was much lower than that of chloramine (above 10 mg/L despite water matrix).

In addition, TEM was used to examine cell membrane damage of the recipient strain under chloramine or free chlorine exposure. It was seen that the cell membranes were distinct in the untreated control (Fig. 4c). However, obvious cell membrane damage was evident when the cells were exposed to 10 mg/L of chloramine or free chlorine (Fig. 4d, e).

Damage of the cell membrane caused by the disinfectants was further assessed by analysis of protein and gene expression in the recipient *A. baylyi* (Tables S7, S8). After the exposure to disinfectants for 2 h, the levels of membrane protein were altered (Fig. 4f). For example, 2.5- and 2.0-fold increases of AdeA protein was observed in response to chloramine or free chlorine exposure, respectively. In particular, OprC and BamE proteins were more abundant under disinfectant exposure. These proteins are important for outer membrane channels. The corresponding genes, such as *bamA* and *bamE*, also showed increased expression in the presence of disinfectants (Fig. 4g). In addition, most genes coding for outer membrane protein had increased expression under the exposure of disinfectants. For example, the major outer membrane protein regulator genes, *ompA* and *ompW*, were both significantly upregulated by 1.4-fold in the recipient bacteria in response to the 10 mg/L chloramine exposure. These outer membrane proteins are important for regulating cell membrane permeability [23, 42]. Genes responsible for the efflux pump [43, 44], *hca* family genes and *acr*, were upregulated as well. In addition, genes related to membrane proteins, such as *slyD*, *fumC* and *tolC*, were also upregulated during the disinfectant exposure. Thus, genes and proteins relevant to the cell membrane permeability and integrity were upregulated following exposure to the disinfectants in System 1.

### Loss of transformation frequency after treating naked plasmids with disinfectants

**Fig. 5:**
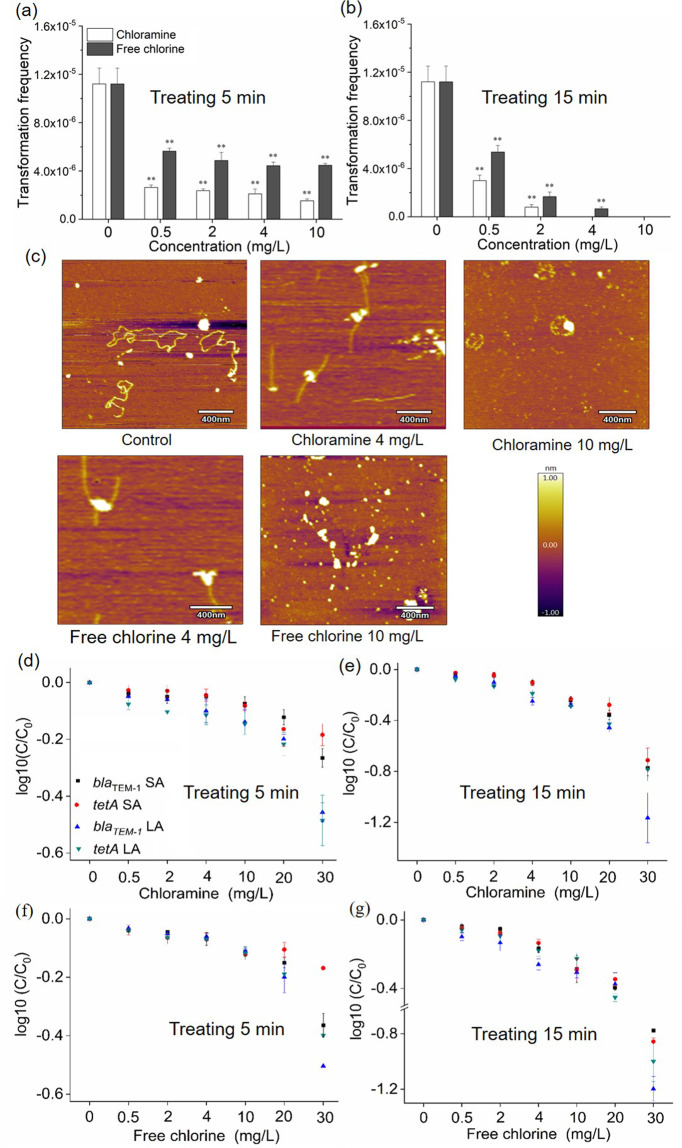
Effects of disinfectant pretreatments on the transformation of pWH1266 plasmid. pWH1266 plasmids pretreated by disinfectants for 5 min (**a**) and 15 min (**b**) caused the decrease in transformation frequency, respectively. **c** AFM images of naked plasmid pWH1266 DNA before and after 15 min of chloramine or free chlorine treatment. The genes *bla*_TEM-1_ and *tetA* degradation versus chloramine treating with 5 min (**d**) and 15 min (**e**) measured with qPCR amplicon. *bla*_TEM-1_ and *tetA* degradation versus free chlorine treating with 5 min (**f**) and 15 min (**g**) measured with qPCR amplicon. The error bars indicate one standard deviation from the mean (*n* ≥ 3). For **a**, **b**, significant differences between individual chloramine or free chlorine and the control were shown with *(*p*_*adj*_ < 0.05) and **(*p*_*adj*_ < 0.01).

In order to test whether the transformation performance of damaged eARGs would be decreased, we set up transformation System 3 (Fig. 1), in which pWH1266 plasmid was first treated with chloramine or free chlorine, prior to being applied to the transformation assay, with doses up to 10 mg/L. Then we measured the ability of eARGs to be acquired by *A. baylyi*. The transformation frequency of eARGs decreased with increasing disinfectant doses (Fig.5a, b). For instance, after treating pWH1266 plasmids with 10 mg/L free chlorine for 5 min, the transformation frequency was 2.5 times lower than that of the control group (Fig. 5a, *p*_*adj*_ = 0.006). In addition, if the pretreatment time was increased to 15 min, no transformants were detected at the exposure of 10 mg/L disinfectants. In this harsh condition, considerable damage to the DNA likely occurred (Fig. 5b). This result is consistent with a previous study where a UV_254_ disinfection treatment could induce transformability loss [6].

To obtain insight into the underlying mechanisms, both AFM and qPCR were employed to explore why the transformation frequency, after the naked plasmid was treated with disinfectants, was decreased. We examined the surface topography of the plasmids before and after chloramine or free chlorine treatments. Circular plasmids were evident in the untreated control (Fig. 5c). However, after exposure to 4 mg/L chloramine or free chlorine, the plasmids were damaged and had become linear. When the dosage of chloramine or free chlorine was increased to 10 mg/L, only short fragments of DNA were observed, instead of intact plasmids (Fig. 5c). These AFM results suggest that the plasmid fragmentation induced by chloramine or free chlorine contributed to the decreased transformation frequency.

Furthermore, we employed qPCR for both short amplicons (SA, ∼200 bps) and long amplicons (LA, 800–1200 bps) to assess the extent of damage of *bla*_TEM-1_ and *tetA* genes before and after the disinfection treatment. As expected, the abundances of both short and long eARGs amplicons decreased as the chloramine or free chlorine doses increased (Fig. 5d–g). At the pretreatment chloramine dose of 30 mg/L for 5 min, the *tetA* LA amplicon abundance decreased by ~0.49 log10 units. In addition, the loss rate of the *bla*_TEM-1_ and *tetA* genes when pretreated with chloramine for 15 min were higher in comparison to those detected when pretreated for 5 min (Fig. 5d, e). For example, at the chloramine dose of 30 mg/L, the abundance of *bla*_TEM-1_ SA genes decreased by about 0.26 log_10_ units after 5 min treatment, whereas this decrease was about 0.77 log_10_ units after 15 min treatment (Fig. 5d, e). Interestingly, for both eARGs, the loss rate of the long gene amplicons was larger than the short gene amplicons (Fig. 5d–g). For example, at the free chlorine dose of 30 mg/L for 5 min treatment, the *tetA* SA was decreased by about 0.17 log_10_ units, whereas the *tetA* LA was decreased by about 0.39 log_10_ units. This is likely due to the larger number of intact short templates of the ARGs existing, in comparison to the long templates for the genes, after the disinfectant treatments. Ultimately, these results confirmed that the damage and loss of eARGs induced by the disinfectants resulted in a decrease of eARGs transformation frequency in System 3.

## Discussion

Disinfection processes such as chlorination are widely used in the final steps of water or wastewater treatment to inactivate pathogens [11–13]. However, these chlorine-based disinfection processes are not able to completely inactivate all of the ARB in treated water or wastewater [45, 46]. Even if disinfection processes could inactivate ARB completely, simultaneously the eARGs could be released into water, thus becoming a source for the spread of ARGs through transformation [47]. Recently, several studies document that disinfection processes have the potential to enhance conjugative transfer [19, 33] or transformation of ARGs [16, 48, 49]. However, no study has evaluated the roles of chlorine-based disinfectants on the dissemination of eARGs by natural transformation comprehensively. Also, it remains unclear as to how and why disinfectants would promote or reduce the spread of ARGs through natural transformation. Here, we established three transformation systems to investigate whether these chlorine-based disinfectants would promote the transformation efficiency of eARGs in conditions mimicking drinking water and WWTP effluent. We report that the transformation frequency of plasmid-encoded ARGs was significantly increased under the exposure of chloramine or free chlorine in both experimental Systems 1 and 2. This is similar to effects detected during antibiotic exposure, where the antibiotics act to increase bacterial ROS generation or cause an SOS response, and thus promote the horizontal transfer of ARGs [3, 50]. In this study, we used multiple approaches (including MICs, plasmid extraction, PCR, and gel electrophoresis) to verify that the pWH1266 plasmid was taken up by *A. baylyi*, and this uptake caused resistance against both Amp and Tet for the recipient. We showed that the disinfectants significantly enhanced the transformation frequency of eARGs at practically relevant concentrations. It should be noted that the recipient *A. baylyi* is detected to be very resistant to disinfectants. Thus, the threshold of disinfectant concentration to promote transformation might vary depending on the types or properties of various recipient strains.

**Fig. 6:**
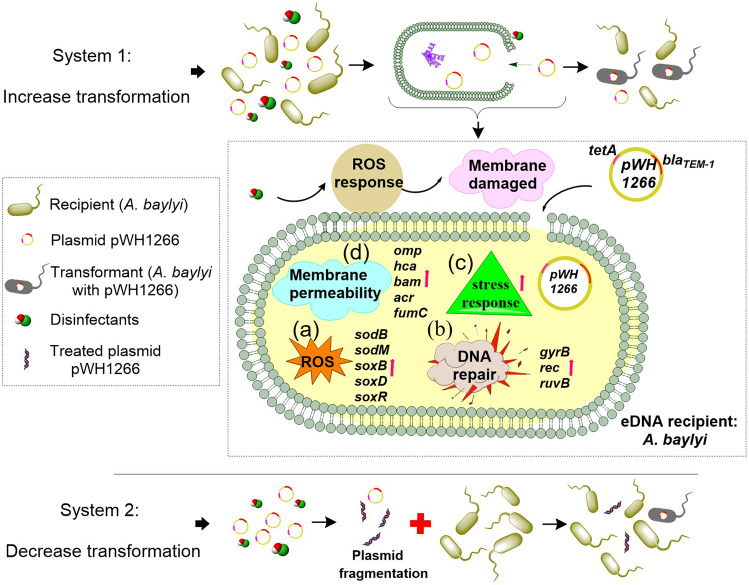
Possible mechanisms that chloramine or free chlorine regulates transformation efficiency of eARGs which are carried on pWH1266 plasmid. Mechanism for System 1: Transformation systems containing the recipient bacteria and plasmid solution, were exposed to different concentrations of disinfectants in mimicking drinking water and mimicking WWTP effluent, respectively. Both chloramine and free chlorine could increase the transformation efficiency of eARGs under the two conditions. **a** Increased intracellular ROS production; **b** Stimulated DNA damage/repair response; **c** Stimulated stress response; **d** Damaged cell membrane. Mechanism for System 3: solutions of purified plasmids were pretreated with disinfectants. Then the treated eARGs were subjected to transformation assays in mimicking WWTP effluent. The damage and loss of eARGs induced by the disinfectants contributed to the decrease of eARGs transformation frequency.

Our studies also sought to determine the underlying mechanisms of how chlorine-based disinfectants promoted the transformation of eARGs thoroughly, by the application of various molecular and visualization approaches. These approaches included the measurement of ROS generation and cell membrane permeability, application of the ROS scavenger test, transformation assays under both aerobic and anaerobic conditions, cell and plasmid morphology characterization by TEM and AFM, genome-wide RNA sequencing and proteomic analysis. The underlying mechanisms we propose for the disinfectant-enhanced transformations of eARGs are summarized in Fig. 6. We expose that the disinfectant exposure induced a series of cellular responses that included the increased generation of ROS, an enhanced cell membrane permeability, an increased stress response and enhanced DNA damage and repair activities.

Among these responses, the increased level of ROS production in *A. baylyi* is a main factor for the disinfectant-caused accelerated transformation of eARGs. This is evident where both the ROS production and transformation frequencies were significantly decreased after the addition of the ROS scavenger. This was consistent with our observations made during disinfectant exposure in anaerobic conditions (Fig. 3b, e), in which no significant enhancement of transformation frequency was detected during exposure to chloramine or free chlorine due to the absence of enhanced ROS generation. Indeed, after the acute exposure to disinfectants for 2 h, the transcriptional levels of oxidative stress (*sodB* and *sodM*) had increased. The transcription levels of stress-related genes (*nirD and glsA*) and competence-related genes (*pilIRTU*) were upregulated under the exposure of 10 mg/L disinfectants (Table S9). In addition to oxidative stress-related genes, DNA integration/repair genes, like *gyrB*, *rec* gene family and *ruvB*, also exhibited increased expression levels. The activated oxidative response was possibly due to DNA damage/repair [16, 51]. Collectively, the evidence compiled of intracellular ROS levels, ROS scavenging, transformation assays under anaerobic conditions, RNA sequencing and protein analyses suggests that generation of intracellular ROS by the cell is physically driving the increased transformation frequency under the exposure of chlorine and chloramine. It should be noted that this study did not validate whether only intracellular ROS or both intracellular and extracellular ROS are playing roles in the increased transformation frequency, which warrants further research. In addition, when exposing *A. baylyi* to chloramine or free chlorine under anaerobic conditions, no significant variations of live/dead cell percentages were seen compared with the non-disinfectant control (Fig. S8). In contrast, under aerobic conditions, high concentrations of chloramine or free chlorine (>20 mg/L) caused significant cell death (*p*_*adj*_ = 2.3 × 10^−4^–4.6 × 10^−2^). This indicates that the disinfection effects are largely resulting from damage caused by increased ROS levels. Consequently, the quenching of ROS may eliminate the antibacterial effects of chloramine and free chlorine. Previous studies also show that microbial ROS production potentiates antibacterial effects [52], and that chlorine-based disinfectants cause a ROS-dependent damage mechanism [53]. However, it should be admitted that there is much controversy about whether ROS changes are symptoms or causes of the increased HGT and antimicrobial induced killing of bacteria [36, 37]. Further studies are required to provide more evidence to prove whether the increased transformation is directly caused by the increased levels of ROS under the exposure of disinfectants.

Increased cell membrane permeability by pore formations on the cell surface may enhance HGT [23, 33]. This present study found that under exposure to chloramine or free chlorine, the cell membrane permeability was significantly increased (Fig. 4a, b, *p*_*adj*_ = 7.2 × 10^−6^–4.9 × 10^−2^). Moreover, the obvious cell membrane damage was observed by TEM when the bacteria were exposed to 10 mg/L chloramine or free chlorine (Fig. 4c). Based on RNA sequencing and proteomic analysis, we found that the disinfectant exposure indeed affected protein abundance and gene expression elements associated with cell membrane integrity and energy production in *A. baylyi*. Moreover, the relationship between cell permeabilization and the increased transformation frequency was evidenced from the observation within System 2, in which *A. baylyi* was pretreated by disinfectants, then the damaged *A. baylyi* was mixed with intact plasmids. Although the intracellular ROS generation dramatically decreased after removing the remaining disinfectants (Fig. S9), the increased cell membrane permeabilization remained. Considering that disinfectant-treated *A. baylyi* possessed a higher ability to uptake eARGs in System 2, these results suggest that the permeable membrane is playing a role in increasing the transformation of eARGs.

In addition, we further revealed the relationship between ROS production and cell permeabilization. Under anaerobic conditions with decreased ROS generation, membrane permeability significantly decreased compared with that under aerobic conditions. For example, the highest fold change was only 2.8-fold under anaerobic conditions (as shown in Fig. S10), while it could reach 40-fold aerobically (Fig. 4a, b). These results suggest there is a close linkage between ROS generation and cell membrane permeability. However, it should also be noted that compared with the non-disinfectant control, a significant enhancement of membrane permeability was shown under the exposure of free chlorine anaerobically (Fig. S10). This might be associated with the fact that disinfectant itself could also pose direct effects on the cell membrane, as reported in a previous study [53].

In contrast to our observations made during disinfectant treatment of the cells, the disinfectant pretreatment of the naked plasmid (i.e., System 3) caused decreased transformation frequency with increasing dosages of chloramine or free chlorine (Fig. 6). Further, if the pretreatment time was prolonged, the naked DNA severely damaged, thus resulting in a significant loss of transformation frequency. By applying AFM and qPCR, we found that the disinfectant exposure fragmented the plasmids and the abundances of the plasmid-borne *bla*_TEM-1_ and *tetA* genes decreased. These results validated that the decrease of eARGs transformation frequency was associated with the plasmid fragmentation induced by the disinfectants (Fig. 6).

The two chlorine-based disinfectants applied in this study could exhibit different modes of action. For example, chlorine forms hydrochloric and hypochlorous acids as well as oxygen radicals, while chloramines decompose to ammonia and hypochlorous acid or hydrochloric acid [54]. A previous study also documents that semi-stable chloramines could even be generated through the reaction of HOCl with DNA, RNA and polynucleotides [55]. After chloramines decay due to metal-ion catalyzed processes, nitrogen-centered radicals could be generated. In this study, we also found that oxygen-based radicals were generated under the exposure of these two disinfectants. However, we did not examine the presence of nitrogen-centered radicals and these may have been generated. It is worthwhile to note that while free chlorine and chloramine are different disinfection agents, they exhibit similar patterns affecting the transformation of eARGs, bacterial ROS generation and membrane permeability. Both free chlorine and chloramine can facilitate transformation mediated by ROS oxidative stress. A major difference in the action of these disinfectants is that the threshold of free chlorine (higher than 0.5 mg/L) causing the increased transformation efficiency is lower than that of chloramines (above 4 mg/L) when *A. baylyi* is the recipient. It will be very relevant and necessary to determine the specific thresholds of chorine and chloramine that can promote the spread of antibiotic resistance in full-scale water or wastewater treatment systems, thus guiding their optimized operation.

Disinfection is an important process to prevent the spread of waterborne pathogens or antibiotic resistance. In this study, we developed three transformation systems to investigate whether practically relevant disinfectant dosages could promote the transformation of eARGs. We found that the chlorine-based disinfectants indeed accelerated the transformation of eARGs by non-ARB. Although eARGs could be damaged by disinfectants in System 3, the transformation ability of eARGs was not completely eliminated. In the current disinfection processes, the levels of bacteria such as *E. coli* are used as indicators to assess the water quality and safety [56, 57]. Our results suggest that the single standard based on pathogen detection might not comprehensively evaluate microbial risks of water safety. Parameters like the presence of iARGs and eARGs could be considered as indicators. Horizontal transmission of iARGs and eARGs needs to be examined in real disinfection processes. In order to comprehensively prevent the transmission of antimicrobial resistance, the current disinfection processes need to be optimized with regard to disinfection concentration, contacting time and disinfectant type. Additionally, new disinfection technologies that can not only inactivate pathogens and ARB, but also damage eARGs and iARGs, should be further developed to minimize the microbial risk [58].

Moreover, during the coronavirus Disease 2019 (COVID-19) pandemic, the use of disinfectants has surged. Consequently, the recorded environmental concentrations of disinfectants have been increasing in the water system through direct discharge of wastewater into receiving waters [59]. As demonstrated in this study and previous studies [16, 19, 33, 49], that disinfectants and disinfection by-products can promote the spread of antibiotic resistance. Thus, the current over-disinfection during the pandemic may have posed an environmental and public health risk by accelerating the spread of antimicrobial resistance, which should be comprehensively evaluated [60].

## Conclusions

This study demonstrated that both chloramine and free chlorine could enhance the transformation efficiency of plasmid-encoded ARGs. Disinfectant exposure induced a series of cell responses, including increased intracellular ROS production, stimulated stress response and increased cell membrane permeability. These changes were accompanied by the accelerated transformation of eARGs. Among these, the oxidative response appears as the most critical factor, as the increased transformation efficiency of eARGs was reversed by the addition of a ROS scavenger or the lack of ROS generation under anaerobic conditions. In addition, these plasmids were fragmented and the ARGs were lost during the exposure of the plasmids to chloramine or free chlorine. The damage and loss of eARGs induced by the disinfectants contributed to the decrease of eARGs transformation frequency. These findings shed light on the roles of commonly used disinfectants in facilitating the horizontal transfer of eARGs in water treatment systems.

## Supplementary information

Supporting information

## Data Availability

RNA sequence data is accessible through Gene Expression Omnibus of NCBI (accession no. GSE142062). The mass spectrometry proteomics data have been deposited in the ProteomeXchange Consortium via the PRIDE partner repository (PXD016794).
