# Supplementary material for: Chlorine disinfection facilitates natural transformation through ROS-mediated oxidative stress
Source: ISME J. 2021 May 3;15(10):2969–85. doi: 10.1038/s41396-021-00980-4 (PMC8091644; doi:10.1038/s41396-021-00980-4)
Supplement: Supplementary file 1 — Supporting information [file 41396_2021_980_MOESM1_ESM.docx]

**Supporting Information**

**Chlorine disinfection facilitates natural transformation through ROS-mediated oxidative stress**

**Running title**: Disinfectants enhance transformation of ARGs

Shuai Zhang^1,2, #^, Yue Wang^1, #^, Ji Lu^1^, Zhigang Yu^1^, Hailiang Song^3^, Philip L. Bond^1,4^ & Jianhua Guo^1^*

^1^Advanced Water Management Centre (AWMC), The University of Queensland, St Lucia, Brisbane, QLD, 4072, Australia

^2^Jiangsu Key Laboratory of Atmospheric Environment Monitoring and Pollution Control, Collaborative Innovation Center of Atmospheric Environment and Equipment Technology, Nanjing University of Information Science & Technology, Nanjing 210044, China

^3^School of Environment, Nanjing Normal University, Jiangsu Engineering Lab of Water and Soil Eco-remediation, Wenyuan Road 1, Nanjing 210023, China

^4^Center for Microbiome Research, School of Biomedical Sciences, Queensland University of Technology, Brisbane, Queensland, 4000, Australia.

^#^ These two authors contributed equally

* Corresponding author. E-mail address: j.guo@awmc.uq.edu.au (J. Guo).

**Text S1. Transformation assays under anaerobic condition**

To further investigate the effects of oxidative stress on disinfectant-mediated transformation, a parallel transformation experiment was conducted with System 1 settings but under anaerobic condition. Briefly, overnight grown *A. baylyi* ADP1 culture was diluted 100 times in LB broth and incubated for 6 h (140 rpm) at 30 °C to reach the OD600 of 1.1. Then cells were collected by 5 min 6,000×g centrifuge for and the pellets were washed twice by using oxygen-free PBS (30 min nitrogen gas bubbling to remove oxygen) in an anaerobic chamber (Coy Laboratory Products Inc., USA). The PBS-washed pellets were then resuspended in oxygen-free PBS containing 50 mg/L COD to reach 10^8^ cfu/mL. 500 *μ*L of the recipient bacteria was mixed with pWH1266 plasmid to reach the final concentration of 0.8 ng/*μ*L. The system was exposed to different concentrations of chloramine or free chlorine (0 (i.e., control), 0.5, 2, 4, 10, 20, and 30 mg/L), respectively, in the anaerobic chamber for 6 h at 25 °C before plating.

**Text S2. Detection of intracellular ROS production in disinfectant-treated *A. baylyi***

For System 2, intracellular ROS production under disinfectants exposure was evaluated after quenching the extracellular disinfectant residue. Firstly, overnight *A. baylyi* ADP1 culture was collected, washed, resuspended in PBS containing 50 mg/L COD. The *A. baylyi* ADP1 was then exposed to different concentrations of chloramine or free chlorine (0 (i.e., control), 0.5, 2, 4, 10, 20, and 30 mg/L), respectively, for 15 min at 25 °C. The samples were centrifuged immediately (12,000×g, 3 min), washed twice and resuspended in 500 *μ*L PBS to remove the extracellular disinfectant. DCFDA was added into the treated *A. baylyi* for the incubation in dark. Afterwards, the intracellular ROS production of treated *A. baylyi* was tested by a CytoFLEX flow cytometer (Beckman Coulter, USA).

**Text S3. Detection of ROS and cell membrane permeability under anaerobic condition**

Intracellular ROS generation and cell membrane permeability of the *A. baylyi* under the exposure of disinfectants were evaluated under anaerobic condition. Briefly, overnight grown *A. baylyi* ADP1 culture was collected, washed, resuspended in oxygen-free PBS containing 50 mg/L COD, which was conducted in an anaerobic chamber (Coy Laboratory Products Inc., USA). For intracellular ROS measurement, the bacterial cultures were stained by DCFDA for 30 min at 37 ^o^C, followed by exposing to different concentrations of chloramine or free chlorine (0 (i.e., control), 0.5, 2, 4, 10, 20, and 30 mg/L) anaerobically. For cell membrane permeability measurement, the bacterial cultures were exposed to different concentrations of chloramine or free chlorine (0 (i.e., control), 0.5, 2, 4, 10, 20, and 30 mg/L) anaerobically, incubated at 25 ^o^C for 6 h, and followed by staining with PI. Both ROS and cell membrane permeability were conducted on a CytoFLEX flow cytometer (Beckman Coulter, USA).


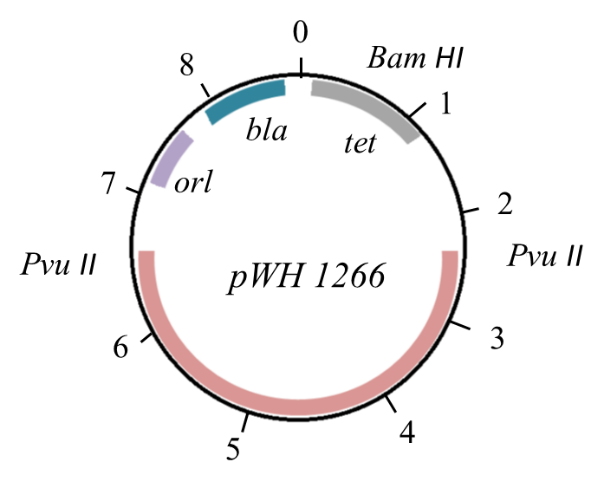


**Fig. S1** Plasmid pWH1266, which is a construct of cryptic plasmid pWH1277 and plasmid pBR322. The cryptic plasmid pWH1277 isolated from *A. colcoaceticus lwoffi* and cloned into the plasmid pBR322. The size of plasmid pWH1266 is 8.89 kb, which carries two ARGs (*tetA* and *bla*_TEM‑1_) against tetracycline (Tet) and ampicillin (Amp).





**Fig. S2** The impacts of time on transformation frequency with plasmid pWH1266 in LB Broth at 25 °C. Experiments were conducted to determine the optimum time to incubate plasmids and bacterial cells prior to plating the bacteria on selective media (n=5). Plasmids (0.8 ng/*μ*L) were added to *A. baylyi* cultures at the log phase (OD600=1.15) and incubated for 2, 4, 6, 8, 12 and 24 h, respectively. Transformation efficiency reached a maximum value (1.5×10^-7^) after 6 h of incubation. Accordingly, a 6 h incubation time was applied for all of the transformation assays. In addition, the OD value remained stable during the treatment process.


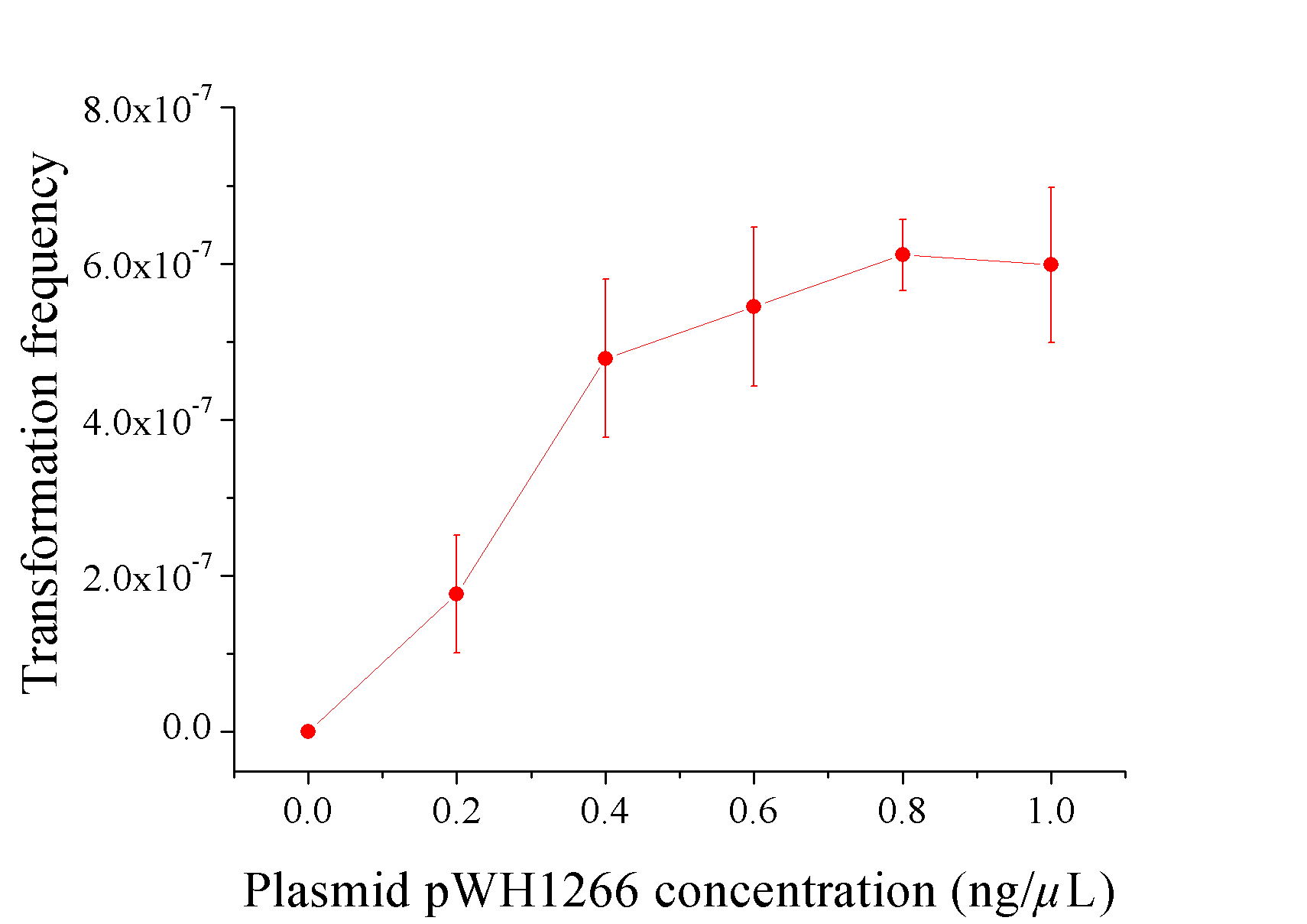


**Fig. S3** Transformation frequency versus the initial plasmid pWH1266 concentration. pWH1266 plasmids were spiked to final concentrations of 0, 0.2, 0.4, 0.6, 0.8 and 1.0 ng/*μ*L in *A. baylyi* cultures (1.15×10^8^ cells/mL), followed by 6-h of incubation at 25 °C. The results suggested that the improvements in transformation efficiency began to level out at plasmid concentrations of 0.8 ng/*μ*L. We therefore employed final plasmid concentrations of 0.8 ng/*μ*L for all the subsequent transformation assays.


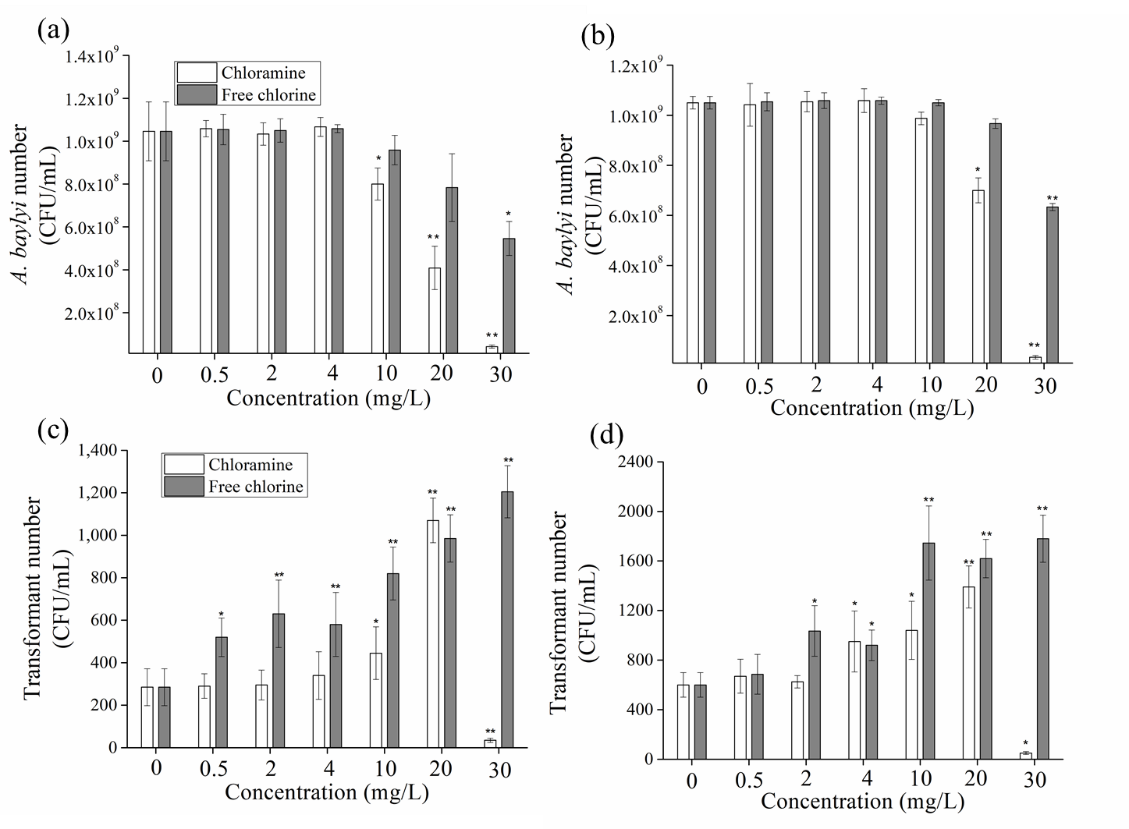


**Fig. S4** Effects of disinfectants on the number of transformant and *A. baylyi* after 6 h exposure in System 1. *A. baylyi* numbers in mimicking drinking water (a) and mimicking downstream of WWTP effluent (b). Transformant numbers in mimicking drinking water (c) and mimicking downstream of WWTP effluent (d). Significant differences between individual chloramine or free chlorine and the control were shown with *(*p_adj_* < 0.05) and ** (*p_adj_* < 0.01).

**
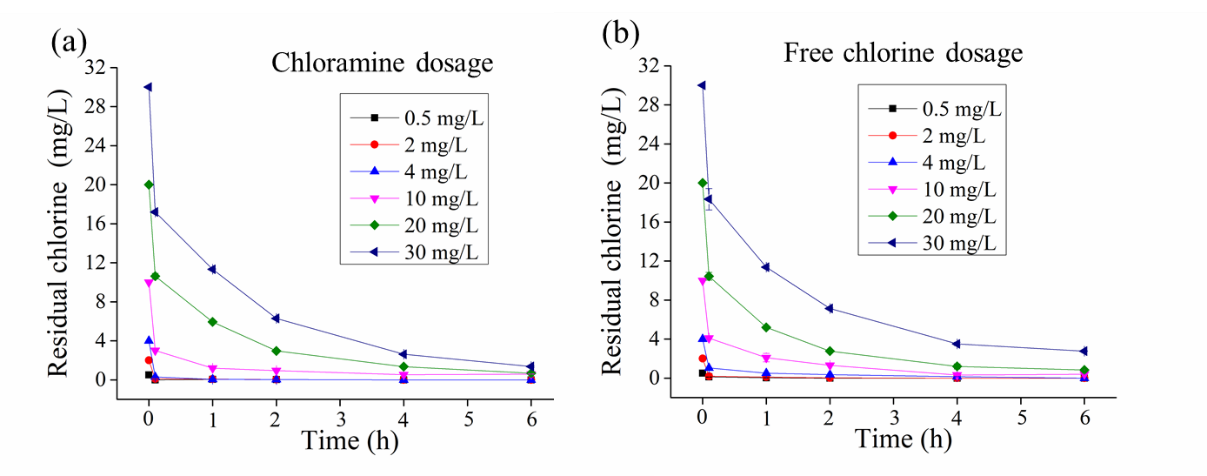
**

**Fig. S5** Variations of residual chlorine during the experiment under mimicking drinking water conditions in System 1. (Data were expressed as mean ± standard deviation based on triplicate experiments)

**Fig. S6** Flow cytometry detection results of intracellular ROS generation when exposing recipient *A. baylyi* to chloramine and free chlorine under aerobic and anaerobic conditions.

**Fig. S7** EPR detection of DMPO-trapped radicals when exposing *A. baylyi* to disinfectants under aerobic and anaerobic conditions. (a) Exposing to 30 mg/L chloramine. (b) Exposing to 30 mg/L free chlorine.

**Fig. S8** Effects of disinfectants on the cell viability of *A. baylyi* under aerobic and anaerobic conditions in System 1. (a) Fold change of live cell percentage comparing with the non-disinfectant control under aerobic condition. (b) Fold change of live cell percentage comparing with the non-disinfectant control under anaerobic condition. (c) Fold change of dead cell percentage comparing with the non-disinfectant control under aerobic condition. (b) Fold change of dead cell percentage comparing with the non-disinfectant control under anaerobic condition.

**

**

**Fig. S9** Fold change of ROS generation of the disinfectant-treated *A. baylyi* (System 2).

**Fig. S10** Fold change of cell membrane permeability of *A. baylyi* under the exposure of disinfectants aerobically and anaerobically.

**Table S1**. Primers selected ARGs used in this study

| Primer | Sequence (5’-3’) | Amplicon Size (bp) | Annealing (℃) |
| --- | --- | --- | --- |
| *blaTEM‑1* Short FW | CGC ACC GGA AAC ATC GCT GCA C | 209 | 53.8 |
| *blaTEM‑1* Short RV | TGA AGT TCC GCC GCA AGG CTC G |  |  |
| *blaTEM‑1* long FW | TCC GGT GGA GGC CGG TAT CTG G | 861 | 51.4 |
| *blaTEM‑1* long RV | CGG GAA TGC CAT CTG CCT TGA G |  |  |
| *tetA* Short FW | TCCGTTCAGCGAATTGGTGCAG | 216 | 53.9 |
| *tetA* Short RV | TTCGTTCACGCCTTACACCAGC |  |  |
| *tetA* long FW | GCTACATCCTGCTTGCCTTC | 1200 | 51.9 |
| *tetA* long RV | CATAGATCGCCGTGA AGAGG |  |  |

**Table S2**. Minimum inhibitory concentrations (MICs) of the recipient *Acinetobacter baylyi* ADP1 and the transformants

| Strains | *A. baylyi* | Transformants* | | | | | | |
| --- | --- | --- | --- | --- | --- | --- | --- | --- |
|  |  | 0 | 0.5 | 2 | 4 | 10 | 20 | 30 |
| TET (mg/L)  AMP (mg/L)  Free chlorine (mg/L)  Chloramine (mg/L) | <5  <50  >100  >50 | >10  >100  >100  >50 | >10  >100  >100  >50 | >10  >100  >100  >50 | >10  >100  >100  >50 | >10  >100  >100  >50 | >10  >100  >100  >50 | >10  >100  >100  >50 |

*: transformants mediated by different concentrations of disinfectants (mg/L).

**Table S3.** The impact of disinfectants (mg/L) on *A. baylyi* ADP1 cultures at the (OD=1.13) and incubated for 6 h.

|  | 0 h | 6 h | | | | | | | |
| --- | --- | --- | --- | --- | --- | --- | --- | --- | --- |
|  | Original value | mg/L | | | | | | | |
|  |  | 0 | 0.5 | 2 | 4 | 10 | 15 | 20 | 30 |
| Free chlorine  (OD_600_ value)  Chloramine (OD_600_ value) | 1.13  1.13 | 1.13  1.12 | 1.13  1.12 | 1.12  1.01 | 1.24  1.07 | 1.14  1.05 | 1.05  1.05 | 1.06  1.05 | 1.03  1.03 |

**Table S4**. Genes relevant to ROS production in *A. baylyi* ADP1 after exposure of chloramine or free chlorine.

| Gene |  | Fold change in FPKM * | |
| --- | --- | --- | --- |
|  | COG Annotation | Chloramine Free chlorine | |
| *rutB* | Pyrimidine utilization protein B | 2.50 | 1.02 |
| *rutC* | Pyrimidine utilization protein C | 2.43 | 1.14 |
| *rutD* | Pyrimidine utilization protein B | 1.73 | 1.13 |
| *sodB* | Superoxide dismutase | 1.84 | 1.37 |
| *sodM* | Superoxide dismutase | 1.31 | 2.39 |
| *soxD* | Sarcosine oxidase subunit beta | 4.72 | 3.81 |
| *soxB* | Sarcosine oxidase subunit beta | 1.78 | 1.36 |
| *alkM* | Alkane 1-monooxygenase | 1.17 | 1.21 |
| *alkK* | Long-chain-fatty-acid--CoA ligase | 1.71 | 2.03 |
| *alkB* | Alpha-ketoglutarate-dependent dioxygenase AlkB | 2.20 | 1.14 |
| *aphC* | Alkyl hydroperoxide reductase subunit C | 4.95 | 1.59 |
| *aphF* | Alkyl hydroperoxide reductase subunit F | 4.31 | 1.04 |
| *soxR* | DNA-binding transcriptional regulator, MerR family | 2.73 | 2.19 |

*: Comparing with the control group without chloramine or free chlorine dosage.

**Table S5**. Genes relevant to DNA integration/repair in *A. baylyi* ADP1 after exposure of chloramine or free chlorine.

| Gene |  | Fold change in FPKM * | |
| --- | --- | --- | --- |
|  | COG Annotation | Chloramine Free chlorine | |
| *dnaA* | Chromosomal replication initiator protein DnaA | 1.45 | 1.30 |
| *dnaN* | DNA polymerase III subunit beta | 1.27 | 1.06 |
| *gyrB* | DNA topoisomerase (ATP-hydrolyzing) subunit B | 1.32 | 1.10 |
| *uvrC* | Excinuclease ABC subunit UvrC | 1.51 | 1.46 |
| *recB* | Exonuclease V subunit beta | 1.14 | 1.02 |
| *recD* | Exodeoxyribonuclease V subunit alpha | 1.54 | 1.49 |
| *recA* | recombinase RecA | 1.11 | 1.09 |
| *dnaE* | DNA polymerase III subunit alpha | 1.29 | 1.37 |
| *recR* | recombination protein RecR | 1.47 | 1.51 |
| *recO* | DNA repair protein RecO | 1.21 | 1.06 |
| *ruvB* | Holliday junction branch migration DNA helicase RuvB | 1.75 | 1.16 |
| *polA* | DNA polymerase I | 1.55 | 1.20 |

*: Comparing with the control group without chloramine or free chlorine dosage.

**Table S6**. Proteins relevant to ROS production in *A. baylyi* ADP1 after exposure of chloramine or free chlorine.

| \| Protein \|  \| Fold change in FPKM * \| \| \| --- \| --- \| --- \| --- \| \| Gene description \| Chloramine Free chlorine \| \| \| SodA \| Superoxide dismutase \| 1.62 \| 2.85 \| \| Dps \| DNA protection during starvation protein \| 1.52 \| 1.32 \| \| KatA \| Catalase peroxidase \| 1.45 \| 1.14 \| \| AhpC \| Alkyl hydroperoxide reductase \| 1.66 \| 1.13 \| \| AphF \| Alkyl hydroperoxide reductase subunit \| 1.04 \| 1.41 \| \| TrxB \| Thioredoxin reductase \| 1.65 \| 1.74 \| \| HimD \| Integration host factor subunit alpha \| 5.31 \| 3.03 \| |
| --- | --- | --- | --- | --- | --- | --- | --- | --- | --- | --- | --- | --- | --- | --- | --- | --- | --- | --- | --- | --- | --- | --- | --- | --- | --- | --- | --- | --- | --- | --- | --- | --- | --- | --- | --- |

*: Comparing with the control group without chloramine or free chlorine dosage.

**Table S7**. Genes relevant to cell membrane in *A. baylyi* ADP1 after exposure of chloramine or free chlorine.

| Gene |  | Fold change in FPKM * | |
| --- | --- | --- | --- |
|  | COG Annotation | Chloramine Free chlorine | |
| *ompA* | Membrane protein | 1.45 | 0.98 |
| *ompW* | Membrane protein | 1.42 | 1.22 |
| *ompH* | Membrane protein | 1.02 | 1.00 |
| *ompR* | Peptidylprolyl isomerase | 1.24 | 0.94 |
| *bamA* | Two-component system response regulator OmpR | 1.00 | 1.28 |
| *acr* | Outer membrane protein assembly factor BamA | 1.21 | 1.46 |
| *bamE* | SDR family NAD(P)-dependent oxidoreductase | 1.15 | 1.96 |
| *hcaA* | outer membrane protein assembly factor BamB | 2.40 | 1.55 |
| *hcaD* | p-hydroxycinnamoyl CoA hydratase/lyase | 1.66 | 1.14 |
| *hcaR* | acyl-CoA dehydrogenase | 2.05 | 1.05 |
| *ACIAD-RS07155* | MarR family transcriptional regulator | 1.75 | 2.82 |
| *hcaR* | MarR family transcriptional regulator | 2.05 | 1.05 |
| *fumC* | MarR family transcriptional regulator | 2.10 | 1.91 |
| *slyD* | Class II fumarate hydratase | 1.35 | 1.81 |
| *lolA* | Peptidylprolyl isomerase | 1.34 | 1.08 |
| *ftsQ* | Outer membrane | 1.31 | 1.37 |
| *tolC* | Outer membrane | 0.98 | 1.20 |
| *ACIAD-RS13665* | TolC family outer membrane protein | 1.22 | 1.48 |
| *ACIAD1549* | MarR family transcriptional regulator | 1.43 | 2.70 |
| *ACIAD2570* | SDR family NAD(P)-dependent oxidoreductase | 1.34 | 1.81 |
| *ACIAD2948* | SDR family NAD(P)-dependent oxidoreductase | 1.01 | 1.40 |
| *ACIAD3555* | SDR family NAD(P)-dependent oxidoreductase | 1.17 | 1.45 |
| *ACIAD0111* | SDR family NAD(P)-dependent oxidoreductase | 3.12 | 3.50 |
| *ACIAD0799* | Membrane protein | 1.61 | 2.34 |
| *ACIAD0898* | Membrane protein | 1.10 | 1.17 |

*: Comparing with the control group without chloramine or free chlorine dosage.

**Table S8**. Proteins relevant to cell membrane in *A. baylyi* ADP1 after exposure of chloramine or free chlorine.

| Protein |  | Fold change in FPKM * | |
| --- | --- | --- | --- |
|  | Gene description | Chloramine Free chlorine | |
| YidC | Membrane protein insertase YidC | 1.34 | 0.83 |
| AdeA | Putative membrane fusion protein | 2.53 | 2.01 |
| BamD | Outer membrane protein assembly factor BamD | 1.27 | 0.76 |
| ACIAD2449 | Putative lipoprotein, attached to the cytoplasmic membrane | 1.04 | 1.09 |
| BamA | Outer membrane protein assembly factor BamA | 0.72 | 1.22 |
| ACIAD1199 | Putative outer membrane W signal peptide protein | 0.95 | 1.27 |
| BamE | Outer membrane protein assembly factor BamE | 1.43 | 1.03 |
| ACIAD0898 | Putative outer membrane protein | 1.75 | 1.74 |
| ACIAD0697 | Putative Outer membrane protein (OmpA-like) | 0.55 | 1.07 |
| BamB | Outer membrane protein assembly factor BamB | 1.09 | 0.80 |
| ACIAD0240 | Putative outer membrane protein | 0.66 | 1.31 |
| OprC | Putative outer membrane copper receptor | 1.56 | 1.01 |
| ComQ | Putative outer membrane protein | 1.04 | 1.16 |

*: Comparing with the control group without chloramine or free chlorine dosage.

**Table S9.** Fold changes of translation of ATP-related genes, stress-related genes, helicase-related genes, and competence-related genes

| Gene | | |  | Fold change in FPKM * | | | |
| --- | --- | --- | --- | --- | --- | --- | --- |
|  |  |  | COG Annotation | Chloramine Free chlorine | | | |
|  | *atpI* | ATP synthase subunit I | | 0.52 | | 0.48 |  |
|  | *atpB* | ATP synthase subunit A | | 0.47 | | 0.45 |  |
|  | *atpE* | ATP synthase subunit C | | 0.67 | | 0.41 |  |
|  | *atpF* | ATP synthase subunit B | | 0.72 | | 0.46 |  |
| ATP | *atpH* | ATP synthase subunit delta | | 0.64 | | 0.60 |  |
|  | *atpG* | ATP synthase subunit gamma | | 0.89 | | 0.63 |  |
|  | *atpD* | ATP synthase subunit beta | | 1.05 | | 0.65 |  |
|  | *atpC* | ATP synthase subunit epsilon | | 1.26 | | 0.77 |  |
|  | *rep* | ATP-dependent DNA helicase | | 1.39 | | 1.16 |  |
|  |  |  |  |  | |  |  |
|  | *ACIAD2014* | Helicase | | 1.04 | | 1.27 |  |
|  | *hrpA* | ATP-dependent RNA helicase HrpA | | 1.27 | | 1.43 |  |
|  |  |  |  |  | |  |  |
| Helicase | *ACIAD2175* | Helicase | | 35.75 | | 46.53 |  |
|  | *ACIAD2185* | DNA helicase | | 25.28 | | 28.44 |  |
|  | *dnaB* | Replicative DNA helicase | | 1.02 | | 1.27 |  |
|  |  |  |  |  | |  |  |
|  | *ruvB* | Holliday junction branch migration DNA helicase RuvB | | 1.75 | | 1.16 |  |
|  |  |  |  |  | |  |  |
|  | *ACIAD1493* | Universal stress protein | | 1.18 | | 1.28 |  |
| Stress | *ACIAD2005* | Universal stress protein | | 1.32 | | 1.13 |  |
|  | *nirD* | NirD/YgiW/YdeI family stress tolerance protein | | 1.16 | | 1.56 |  |
|  |  |  |  |  | |  |  |
|  | *glsA* | Glutaminase | | 1.08 | | 1.05 |  |
|  | *pilR* | Fis family transcriptional regulator | | 1.37 | | 1.28 |  |
| Competence | *pilI* | Purine-binding chemotaxis protein | | 1.49 | 1.51 | |  |
|  | *pilU* | pilU family type 4a pilus ATPase | | 1.23 | 1.43 | |  |
|  | *pilT* | IV pilus regulator protein | | 1.85 | 1.32 | |  |

*: Comparing with the control group without chloramine or free chlorine dosage.

**References**

1. Lu JY, Wang XM, Liu HQ, Yu HQ, Li WW. Optimizing operation of municipal wastewater treatment plants in China: The remaining barriers and future implications. Environ Int. 2019;129:273-278.

2. Wang Y, Lu J, Mao L, Li J, Yuan Z, Bond PL, Guo J. Antiepileptic drug carbamazepine promotes horizontal transfer of plasmid-borne multi-antibiotic resistance genes within and across bacterial genera. ISME J. 2019;13:509-522.
